# From metabolism to malignancy: the multifaceted role of PGC1α in cancer

**DOI:** 10.3389/fonc.2024.1383809

**Published:** 2024-05-07

**Authors:** Yue Wang, Jianing Peng, Dengyuan Yang, Zhongjie Xing, Bo Jiang, Xu Ding, Chaoyu Jiang, Bing Ouyang, Lei Su

**Affiliations:** ^1^ Department of Surgery, Nanjing Central Hospital, Nanjing, China; ^2^ Division of Biosciences, University College London, London, United Kingdom; ^3^ Department of General Surgery, Nanjing Drum Tower Hospital, Clinical College of Nanjing Medical University, Nanjing, China; ^4^ Department of General Surgery, Affiliated Drum Tower Hospital, Medical School of Nanjing University, Nanjing, China

**Keywords:** PGC1α, tumor progression, cancer metabolism, signaling pathways, therapeutic target, metabolic heterogeneity

## Abstract

PGC1α, a central player in mitochondrial biology, holds a complex role in the metabolic shifts seen in cancer cells. While its dysregulation is common across major cancers, its impact varies. In some cases, downregulation promotes aerobic glycolysis and progression, whereas in others, overexpression escalates respiration and aggression. PGC1α’s interactions with distinct signaling pathways and transcription factors further diversify its roles, often in a tissue-specific manner. Understanding these multifaceted functions could unlock innovative therapeutic strategies. However, challenges exist in managing the metabolic adaptability of cancer cells and refining PGC1α-targeted approaches. This review aims to collate and present the current knowledge on the expression patterns, regulators, binding partners, and roles of PGC1α in diverse cancers. We examined PGC1α’s tissue-specific functions and elucidated its dual nature as both a potential tumor suppressor and an oncogenic collaborator. In cancers where PGC1α is tumor-suppressive, reinstating its levels could halt cell proliferation and invasion, and make the cells more receptive to chemotherapy. In cancers where the opposite is true, halting PGC1α’s upregulation can be beneficial as it promotes oxidative phosphorylation, allows cancer cells to adapt to stress, and promotes a more aggressive cancer phenotype. Thus, to target PGC1α effectively, understanding its nuanced role in each cancer subtype is indispensable. This can pave the way for significant strides in the field of oncology.

## Introduction

The peroxisome proliferator-activated receptor gamma coactivator 1-alpha (PGC1α) is a pivotal transcriptional coactivator with multifaceted roles in regulating cellular energy metabolism and mitochondrial biogenesis (1). Since its first identification as a binding partner of the nuclear receptor peroxisome proliferator-activated receptor gamma (PPARα) in 1998, the significance of PGC1α in orchestrating diverse metabolic pathways has become increasingly evident (2, 3). Its functions include mitochondrial biogenesis, fatty acid oxidation, gluconeogenesis, and oxidative phosphorylation (4).

The versatility of PGC1α is emphasized by its ability to interact with a multitude of transcription factors and coactivators (5, 6). This enables PGC1α to exert intricate control over the expression of genes relevant to these pathways. As the cornerstone of mitochondrial biogenesis (7), PGC1α can augment the number and activity of mitochondria. This amplification enhances cellular energy production and adaptability, ensuring a balance between energy homeostasis and the response to shifts in energy demands. Beyond its metabolic functions, PGC1α is pivotal in processes such as cell growth, differentiation, and survival (8), underscoring its role in preserving cellular integrity and function. The prominence of PGC1α in physiological processes implies its potential involvement in pathologies. Indeed, perturbations in PGC1α expression or activity have been linked to a spectrum of diseases, ranging from neurodegenerative disorders (9) and metabolic syndromes to cardiovascular diseases (10). Notably, emerging evidence suggests a role for PGC1α in cancer pathogenesis (11). Its dysregulation has been implicated in metabolic reprogramming and disease progression across a variety of malignancies, both solid and hematological (1).

Unfortunately, challenges exist in managing the metabolic adaptability of cancer cells and refining PGC1α-targeted approaches. There is a need to decode the intricate molecular ties of PGC1α’s interactions with cancer cells. This is a critical step in comprehending both PGC1α’s multifaceted involvement in cancer and the ability to use this knowledge for cancer prevention and treatment. Therefore, this review aims to collate and present the current knowledge on the expression patterns, regulators, binding partners, and roles of PGC1α in diverse cancers. We examined PGC1α’s tissue-specific functions and elucidated its dual nature as both a potential tumor suppressor and an oncogenic collaborator. A comprehensive understanding of PGC1α’s intricate relationship with cancer metabolism could pave the way for novel biomarker identification and therapeutic interventions against this pervasive global health challenge. We hope that this review will serve as a foundational guide for researchers interested in the further exploration of this domain.

## Structure and functions of PGC1α

The PGC1α gene, located on chromosome 4 at the 4p15.1 position, features several functional domains crucial for its activity (3). These domains include the N-terminal transcriptional coactivatory domains, which facilitate interactions with various transcription factors; a central inhibitory domain that moderates its coactivatory functions; and an RNA recognition motif (RRM) located at the C-terminus. The N-terminal region is particularly significant for its role in engaging with nuclear receptors such as peroxisome proliferator-activated receptor gamma (PPARγ), nuclear respiratory factor 1 (NRF1), and estrogen-related receptor alpha (ERRα) (1, 12). These interactions are essential for the transcriptional regulation of genes that play a role in mitochondrial functionality and oxidative metabolism. Upon activation, PGC1α primarily functions by coactivating nuclear receptors and other transcription factors, thereby promoting the expression of genes related to energy metabolism (13). This gene is fundamental to the adaptation to changing metabolic demands during shifts in nutrient availability or energy requirements. Specifically, PGC1α enhances mitochondrial replication, respiratory capacity, and oxidative phosphorylation, which collectively increase cellular energy production (14). The regulation of its activity involves various post-translational modifications and protein interactions, allowing for a responsive adjustment to cellular energy conditions (15, 16). Through these multifaceted roles and complex regulatory mechanisms, PGC1α acts as a central regulator of metabolic processes, highlighting its importance in both normal physiology and various pathological conditions, including cancer.

## Roles of PGC1α in cancer cells

**Figure 1:**
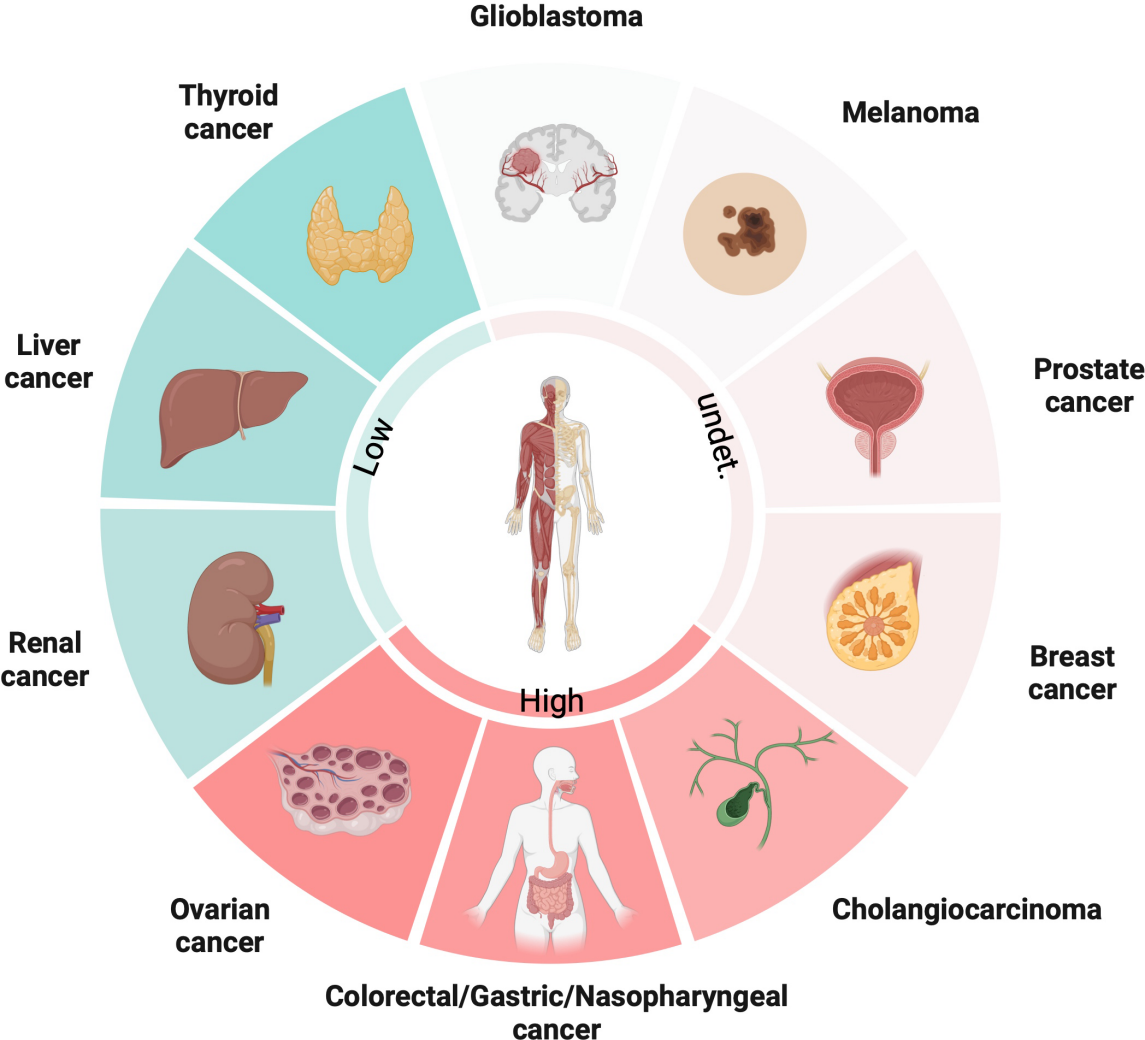
The expression patterns of PGC1α in diverse cancers.

PGC1α is an important gene that regulates metabolism in cancer cells, controlling pathways like glycolysis, tricarboxylic acid (TCA) cycle and fatty acid synthesis etc. But the interesting thing is its actual role differs a lot between different cancer types – sometimes it suppresses tumors, but other times it promotes cancer growth instead! This depends on the complex interplay between various intracellular signaling pathways. PGC1α closely interacts with molecules like β-catenin, AMP-activated protein kinase (AMPK), and can modulate downstream gene expression based on these upstream signals. So, it acts like a central integrator of signals that reprogram metabolism. Its expression and functions are super tissue-specific. For example, from the latest studies, PGC1α expression is upregulated in ovarian cancer (OC), colorectal cancer (CRC), gastric cancer (GC), nasopharyngeal (NPC) and cholangiocarcinoma (CCA), while downregulated in thyroid cancer (TC), liver cancer and renal cancer. However, in some types of cancers, like melanoma, prostate and breast cancer, low and high expressions of PGC1α are coexisted (**Figure 1**). PGC1α also reshapes the tumor microenvironment by coordinating metabolic crosstalk between cancer cells and immune cells. Targeting PGC1α could potentially help overcome therapeutic resistance, but overcoming metabolic plasticity of cancer cells remains a big challenge! Here, we elucidate the mechanisms of PGC1α from the perspective of different tumor cells in detail.

### PGC1α in thyroid cancer

The conventional perspective on metabolic changes observed in thyroid carcinomas is that they arise as a consequence of disease progression rather than as instigators themselves (17, 18). However, growing evidence suggests that these metabolic alterations also play regulatory roles in driving cancer progression. Studies have revealed the downregulation of PGC1α expression, particularly in the advanced stages of thyroid cancer (19) and notably in tumors harboring the BRAF V600E mutation (17), which is the most prevalent somatic oncogenic mutation in papillary thyroid carcinoma (20, 21). A comprehensive analysis on The Cancer Genome Atlas (TCGA) data from hundreds of patients with papillary thyroid carcinoma underscored the significance of PGC1α downregulation, as it correlated with a higher disease stage and an increased risk of recurrence (17). Multiple mechanisms appear to be involved in suppressing PGC1α expression in TC. The intricate AMPK signaling pathway is implicated in this regulatory process, whereby the activation of protein kinase B (AKT) leads to the suppression of PGC1α expression (17). Further, oxidative metabolism appears to inflict damage upon PGC1α mRNA, consequently dampening its expression (17). This forms a vicious feedforward loop as PGC1α loss exacerbates oxidative stress by curtailing mitochondria and antioxidant responses. Given multifaceted aspects of negative regulation, PGC1α is capable of playing important roles in TC development. Indeed, PGC1α deficiency damages mitochondrial function, elevates oxidative stress, and enhances glycolytic phenotype and disease progression (17).

### PGC1α in colorectal cancer

PGC1α is frequently found to be overexpressed in CRC tissues and cell lines. It serves as a key energy mediator, and is induced by aerobic glycolysis (22) and hypoxia (23) in CRC cells. Interestingly, lactate metabolites generated from these processes contribute to the elevation of PGC1α mRNA levels in cancer cells (24, 25). Sirtuin3 (SIRT3), a principal mitochondrial deacetylase, has been implicated in modulating PGC1α levels. In metastatic CRC cell lines, the inhibition of SIRT3 using shRNAs has been found to lead to a decrease in both PGC1α mRNA and protein levels (26). Additionally, oncogenic phosphatase PRL3, a regulator of reactive oxygen species (ROS), has been found to exert its influence on upregulated PGC1α expression through the mediation of Ras-proximate-1(RAP1) (27). However, in specific scenarios where CRC cells are quiescent, the level of PGC1α has been found to be reduced. For example, linoleic acid was found to induce dormancy in CRC cells by increasing the expression of miR-494, which suppressed energy metabolism genes and maintained cell quiescence through reducing PGC1α levels (28). Interestingly, miR-494 was present at low levels in non-metastatic cases (28). In addition, in normoxic CRC cancer stem cells (CSCs), the expression of hypoxia-inducible factor 1-alpha (HIF-1α) was significantly reduced, leading to the restoration of PGC1α expression in these areas (29). This suggests that HIF1α can act as a negative regulator of PGC1α. Additionally, substances secreted from the tumor microenvironment (TME) may also impact PGC1α expression. For instance, neutrophil elastase (NE), which is released from the neutrophil extracellular traps (NETs) activated toll-like receptor 4 (TLR4) on cancer cells, has been shown to trigger the TLR4/p38/PGC1α axis, resulting in PGC1α upregulation (30). Notably, PGC1α levels have been found to not only be upregulated in cancer cells but also be heightened in adipose tissues (20), particularly in the context of obesity-related CRC (31) and in cases of cancer cachexia (32). This cachexia-related elevation of PGC1α has been shown to be achieved through the secretion of interleukin-8 (IL-8) in extracellular vesicles (EVs) from CRC cells (32). Cachexia, a late-stage complication of various cancers, is promoted by tumor growth and chemotherapy administration (33). In muscle tissues affected by wasting, abnormal PGC1α expression contributes to mitochondrial dysfunction, which in turn causes cachexia-related symptoms (34). Retrospective studies analyzing clinical tumor samples from patients with CRC have underscored the clinical relevance of PGC1α in CRC (35). Notably, PGC1α expression is positively correlated with nodal metastasis (36), and high tumor PGC1α expression is correlated with reduced overall survival (OS) (36). Hence, PGC1α might serve as a biomarker for assessing CRC invasion and progression.

Functionally, PGC1α plays a crucial role in enhancing mitochondrial biogenesis and oxidative phosphorylation, thereby reprogramming the metabolism to support cell proliferation, growth, and survival (23, 30, 37). Particularly under hypoxic conditions, PGC1α exerts an antioxidant effect to shield cancer cells from accumulation of ROS (23). Moreover, PGC1α enhances lipid biosynthesis by increasing fatty acid synthase (FASN) levels indirectly through the upregulation of Sp1 and SREBP-1c. This process provides essential building blocks for cell membranes in rapidly proliferating cells (38). Further, PGC1α activates multiple pro-tumorigenic signaling pathways in CRC. It promotes the activation of the AKT/GSK-3β pathway through physical interactions with AKT, although the exact mechanisms remain undefined (39). Additionally, PGC1α boosts leucyl-tRNA synthetase 1 (LARS1) expression, further stimulating AKT/GSK-3β signaling (37).WNT/β-catenin pathway can also be activated by PGC1α, which promotes CRC cell proliferation and inhibits apoptosis (37, 39). PGC1α activates the epithelial-mesenchymal transition (EMT) pathway by upregulating transcription factors like Snail, Slug, and Twist (37, 39), thereby facilitating cancer cell migration and invasion. Recent findings have also indicated that PGC1α can orchestrate lactate oxidation, further promoting the migration and invasion of normoxic CSCs in CRC (29). Additionally, PGC1α can upregulate oxidative phosphorylation and antioxidant genes in chemo resistant cells to adapt to metabolic stress and evade damage from chemotherapeutic agents (23, 40). In 5FU-resistant CRC cells, elevated PGC1α expression has been found to be associated with enhanced mitochondrial biogenesis (40), increased expression of BCL2 while simultaneous decreases BAX, cleaved caspase-3, and cleaved PARP-1 (23). Although SIRT1 is not found to regulate PGC1α transcriptionally, it controls deacetylation and activation of PGC1α, thus protecting CRC cells against chemotherapy (41). Suppression of PGC1α restores chemosensitivity in CRC cells (41). Consequently, monitoring changes in PGC1α expression in patient samples during and after treatment can provide insights into tumor response and the progression towards chemoresistance, enabling timely adjustments to alternative treatment regimens before the disease advances.

Above all, PGC1α integrates various oncogenic pathways in CRC, including metabolism, EMT, inflammation, and survival. Targeting PGC1α holds promise as an approach to counter metastasis and improve patient outcomes. Importantly, dietary interventions offer a potential strategy. For instance, linoleic acid (LA) has been shown to induce quiescence in CRC by suppressing PGC1α expression in mice models (28). *In vitro* studies have demonstrated that manuka honey (MH) reduces colon cancer cell growth in a dose-dependent manner by deactivating PGC1α (42). Clinical trials for CRC are also exploring metabolic drugs such as metformin, which indirectly inhibit PGC1α activity and reprogram metabolism (11, 43). Given their favorable safety profiles and ability to target cancer metabolism, these PGC1α-related drugs could offer new treatment avenues for patients with advanced or chemotherapy-refractory CRC.

### PGC1α in gastric cancer

Mirroring its expression pattern in CRC, PGC1α has been found to be highly expressed in GC and gastric epithelial cells (44). This elevated expression appears to be influenced by oxidative stress induced by exogenous molecules. Indeed, research has demonstrated that quercetin, a potential prooxidant, increases PGC1α expression under oxidative stress conditions, thereby safeguarding gastric cells from damage (45). Notably, this effect is particularly pronounced after prolonged exposure to H_2_O_2_, whereas quercetin lacks this effect under normal circumstances (45).

Upregulated PGC1α has been found to promote GC progression through the inhibition of cell apoptosis and promotion of EMT (44). A more precise mechanism has now been revealed, wherein PGC1α orchestrates the transcription of SNAI1, subsequently affecting the levels of miR-128b (44). This regulatory cascade, which has been observed both *in vitro* and *in vivo*, enhances cell growth and metastasis in GC (44). The influence of posttranscriptional modifications on PGC1α’s function is also notable. In GC, PGC1α has been found to undergo phosphorylation by CAB39L-induced p-AMPK, culminating in the regulation of genes associated with mitochondrial respiration complexes (46). Furthermore, PGC1α possesses a pivotal role in the chemoresistance of GC, characterized by disrupted metabolism (47, 48). The HCP5/miR-3619-5p axis controls PGC1α expression, subsequently enabling its interaction with CCAAT/enhancer binding protein beta (CEBPB), thereby triggering transcription of carnitine palmitoyltransferase I (CPT1). This in turn enhances fatty acid oxidation (FAO) in GC, ultimately conferring chemoresistance to cancer cells (49). Remarkably, PGC1α suppression could sensitize GC cells to chemotherapeutic agents by inducing metabolic deficiencies and increasing oxidative stress (49).

### PGC1α in liver cancer

Liver cancer, also known as hepatocellular carcinoma (HCC), is a major cause of cancer-related mortality worldwide (50). The main risk factors for HCC include chronic hepatitis B and C viral infections, alcohol abuse, nonalcoholic steatohepatitis (NASH), and aflatoxin exposure (51, 52).

Emerging evidence suggest that PGC1α is downregulated in HCC tissues and cell lines (2, 53). However, the cause of the abnormal expression remains unclear. One proposed mechanism is the accumulation of Parkin-interacting substrate (PARIS) in response to oxidative stress (54), which inhibits PGC1α expression transcriptionally. Additionally, sestrin2 (SESN2), a stress-inducible protein in HCC, has been found to mediate glutamine-dependent activation of PGC1α. SESN2 forms a complex with JNK and FOXO1, enhancing PGC1α transcription. Thus, the reduced SESN2 leads to a decreased PGC1α expression under glucose deprivation (55). The Yes-associated protein 1 (YAP1), a key effector of the Hippo signaling pathway, suppresses PGC1α expression in HCC (56). Moreover, mitochondrial transcription factor B2 (TFB2M), acts as a pivotal oncogene in HCC (57), decreases PGC1α expression at both mRNA and protein level through SIRT3/HIF-1α signaling (58). Interestingly, although hypoxia generally induces PGC1α expression in many diseases (59–61), HIF-1α has been reported to negatively regulate PGC1α expression (58, 62). However, the precise mechanism underlying this regulation remains unclear, due to a lack of Chip-Seq data and in-depth investigations.

Functional studies have demonstrated that PGC1α inhibits HCC cell proliferation and metastasis. Low PGC1α expression is associated with poor prognosis and aggressive tumor features in HCC patients (2). Mechanistically, PGC1α has been found to counter the Warburg effect, a well-known process promoting cancer progression in HCC cells (63, 64). PGC1α achieves this by promoting oxidative phosphorylation (OXPHOS) and inhibiting aerobic glycolysis, partially through PDK1 in a PPARγ-dependent manner (2). PGC1α activates PPARγ, leading to reduced β-catenin protein levels and inhibition of the WNT/β-catenin pathway and PDK1 expression (2). This results in a decrease in the Warburg effect and tumor suppression. Conversely, impaired PGC1α reverses these effects (2). Moreover, PGC1α regulates gluconeogenic genes with several coactivators, such as hepatic nuclear factor 4 alpha (HNF4α), which has been found to repress pathogenesis of HCC (65). Important targets like G6PC and PCK1, affecting the glycogen accumulation and driving HCC progression (66), are mediated by PGC1α and HNF4α. YAP reduces the ability of PGC1α to coactivate HNF4α at its promoter (56). Post-translational modifications of PGC1α also plays roles in HCC progression. Mitochondrial fission, important in promoting tumor progression in HCC (67, 68), reduces NAD^+^ levels and SIRT1 activity, leading to increased acetylation of PGC1α protein. Reduced PGC1α activity has been found to be associated with downregulation of CPT1A and acyl-CoA oxidase 1 (ACOX1) and inhibition of FAO in HCC cells (53), both contributing to HCC growth and metastasis (69, 70). Interestingly, general control non-depressible 5 (GCN5) has been found to inhibit PGC1α activity via acetylation (16), but the knockout of GCN5 in mouse liver has not been found to have a significant effect on cancer development (71).

PGC1α also has implications in precancerous or tumorigenic stages. High mobility group AT-hook 1 (HMGA1), a non-histone nuclear protein (72), has been found to recruit protein PGC1α to enhance HBV replication and antigen production through HBV EII/Cp promoter activation, which is associated with liver cirrhosis and HCC oncogenesis (73–75). PGC1α’s relationship with viral expression of HBV has also been observed in other studies (76, 77). Liver cirrhosis, stemming from non-alcoholic fatty liver disease (NAFLD), occasionally precedes HCC and involves Bcl-3 (78), while Bcl-3 reduces PGC1α activity, suggesting that higher PGC1α activity might protect against NAFLD-related liver cirrhosis (78, 79). Indeed, pharmacologically activating PGC1α has shown promise for NAFLD treatment (78, 80).

However, several studies have proposed an oncogenic function for PGC1α downregulation validations in HCC. For instance, SET8 inhibits Keap1 expression through PGC1α, activating the Nrf2/ARE pathway and supporting HCC progression (81, 82). Interestingly, gankyrin elevates TIGAR level, a well-known regulator of glucose metabolism, via the Nrf2/ARE pathway. This elevation promotesPGC1α nuclear importation, and drives increased glucose metabolism in HCC (83). Therefore, the synergy between Nrf2/ARE activation and nuclear localization of PGC1α could serve as a critical loop in the metabolic changes that support HCC progression. In addition, the phosphoserine aminotransferase 1 (PSAT1)’s interaction with p53^72P^ variant in HCC cells dissociates PGC1α binding, promotes PGC1α’s nuclear translocation (84), mitochondrial transcription factor A (TFAM)-mediated OXPHOS and TCA cycle activation (15). Converse effects have been observed for wild-type p53. In HCC, CD147 promotes p53 degradation via the PI3K/AKT pathway (85). Intriguingly, when p53 is exogenously expressed, there is an upregulation of PGC1α levels (86). This suggests that CD147 might impede mitochondrial biogenesis and functionality by suppressing PGC1α/TFAM levels. In this context, PGC1α appears to play a tumor-suppressive role. Thus, targeting PGC1α upstream or downstream pathways is a promising therapeutic strategy for HCC. PPARα agonists that mimic PGC1α re-expression have shown efficacy in HCC models. For example, GW7647 diminishes hepatocarcinogenesis in-humanized mice models (87, 88).

### PGC1α in renal cancer

Research has indicated a decline in PGC1α expression in clear cell renal cell carcinoma (ccRCC) tumors compared to normal tissues. This reduction in PGC1α levels aligns with higher tumor grades (89), advanced disease stage (90), worse disease progression, and worse OS (91). One possible reason for this suppression could be the activation of transforming growth factor beta (TGF-β) signaling, which is commonly observed in ccRCC. In fact, when TGF-β signaling is inhibited, PGC1α levels see an increase (92). Moreover, histone deacetylase 1 (HDAC1) and histone deacetylase 7 (HDAC7) have been identified as corepressors, playing a role in suppressing PGC1α via the TGF-β signaling pathway (92). In a related observation, retinoic acid 13 (Stra13 or Dec1) is found to transcriptionally inhibit PGC1α expression. This suggests that Stra13 could be a mediator of HIF-mediated PGC1α suppression during von Hippel-Lindau (VHL) deficiency and hypoxia in ccRCC (90). The actions of Stra13 appear to be closely related to HDAC activity (93). On another front, the epigenetic changes also seem to play a part, particularly through m6A modifications that impacts the stability of PGC1α. A decrease in FTO expression in ccRCC has been linked to a rise in methylated PGC1α mRNA, leading to reduced stability (91).

Functional experiments have uncovered PGC1α’s potential as a tumor suppressor in ccRCC. Evidence suggests that reintroducing PGC1α restores the levels of TCA cycle enzymes and mitochondrial functions, reversing the metabolic effects of TGF-β signaling in mice models (92). In addition to inducing oxidative stress, PGC1α sensitizes ccRCC cells to cytotoxic therapies (90). Moreover, PGC1α inhibits cell metastasis *in vitro* and *in vivo* by reducing collagen gene expression via miR-29a induction, including collagen type I alpha 1 chain (COL1A1) and collagen type VI alpha 2 chain (COL6A2) (94). Loss of PGC1α in metastatic RCC promotes collagen expression, discoidin domain receptor tyrosine kinase 1 (DDR1) activation, and subsequent snail family transcriptional repressor 1 (SNAIL) stabilization (89). Another tumor suppressor, mitochondrial pyruvate carrier 1 (MPC1), is also regulated by PGC1α (95, 96). PGC1α stimulates the transcription of MPC1 in conjunction with ERR-α and reduced MPC1 negates PGC1α’s effects on mitochondrial respiration and biogenesis (95).

However, PGC1α’s role seems subtype-dependent. Divergent conclusions have been drawn for the other subtypes. One instance involves the loss of MYBBP1A in 9% of renal tumors (97). MYBBP1A represses PGC1α levels, so the decline of MYBBP1A activates PGC1α directly and indirectly through c-MYB, shifting cellular metabolism from glycolysis to OXPHOS (98). This occurs primarily in the absence of c-MYB or pVHL (97, 98). Another scenario involves inactivation of SETD2 in approximately 12% of ccRCC cases (99). SETD2, a histone H3 lysine trimethyltransferase, acts as a ccRCC tumor suppressor (100, 101). Loss of SETD2 boosts PGC1α expression and mitochondrial mass in ccRCC (102), prompting a metabolic shift towards oxidative phosphorylation and lipogenesis. In both these contexts, PGC1α takes on a tumor-promoting role in ccRCC.

### PGC1α in cholangiocarcinoma

The metabolic reprogramming observed in CCA plays a crucial role in driving its progression (103, 104). CCA cells exhibit increased aerobic glycolysis and glutamine anaplerosis, which allows them to produce essential biosynthetic intermediates vital for their rapid growth and survival (103). Recently, the significance of PGC1α in CCA has been emphasized. Patients with elevated levels of PGC1α expression tend to experience reduced OS and progression-free survival (PFS) and are associated with increased angioinvasion and accelerated recurrence (105). Furthermore, the upregulation of PGC1α drives CCA metastasis by elevating the expression of two critical factors: pyruvate dehydrogenase-alpha 1 (PDHA1) and mitochondrial pyruvate carrier 1 (MPC1) (96). This molecular mechanism reverses the Warburg effect, a hallmark metabolic characteristic often observes in cancer cells. Notably, PGC1α also exerts a significant influence on mitochondrial metabolism regulation and the maintenance of stem-like characteristics in CCA stem cells (105). Therefore, pharmacological interventions involving substances like metformin or SR-18292 have shown promise in inhibiting the effects associated with PGC1α upregulation, mitigating its impact on CCA progression and metastasis (105).

### PGC1α in glioblastoma

Emerging evidence underscores the pivotal role of PGC1α in GBM oncogenesis, progression, and treatment resistance. Notably, data from the GBM TCGA and GBM PDX Mayo Clinic databases indicates that GBM exhibits decreased PGC1α mRNA expression compared to normal brain tissue (106). Intriguingly, protein levels of PGC1α have also been reported to be highly expressed in GBM patients, which are located not only in the perinuclear or cytoplasmic regions but also prominently within mitochondria, as proven by publicly available TMAs from US Biomax (107). However, compared to WHO grade IV gliomas, lower-grade gliomas (WHO grade II and III) show increased expression of PGC1α (108). Further survival analysis have indicated that higher PGC1α expression in patients with GBM corresponds to shorter survival times (108), implying that PGC1α loss contributes to gliomagenesis and the transition to glioblastoma. Once a GBM develops, the upregulation of PGC1α within a subset of tumors can promote aggressiveness by driving mitochondrial metabolism. Interestingly, the expression of PGC1α varies in distinct PTEN status; therefore, the protein levels of PGC1α are highest in the SF767 cells (PTEN wildtype) and lowest in the A172 cells (PTEN-deleted) (109).

Functional studies demonstrate that PGC1α seems to act as a tumor suppressor in GBM. Aurora kinase A (AURKA) has been implicated in GBM progression and is a potential therapeutic target for this aggressive brain cancer (106, 110). Research has shown that the inhibition of AURKA leads to c-Myc suppression, subsequently resulting in the upregulation of PGC1α, which in turn promotes oxidative metabolism. Furthermore, H3K27ac ChIP-seq and ATAC-seq show that chromatin accessibility at the potential c-Myc-binding region in the PGC1α promoter is increased, whereas following AURKA inhibition, the binding of c-Myc to the PGC1α promoter is reduced. Concurrently, an enhanced acetylation of the same region in PGC1α promoter has been observed following exposure to AURKA inhibition, indicating that c-Myc may act as a suppressor of PGC1α (106). Moreover, FDA-approved HDAC inhibitors, such as panobinostat, vorinostat, and romidepsin, have been shown to replicate these effects by blocking the Warburg effect in GBM cells. This interference with HDAC1/-2 reduces c-Myc levels while increasing PGC1α expression (111). Another inhibitor, crizotinib, which targets MET kinase (112, 113), induces the metabolic reprogramming of GBM cells. This reprogramming, characterized by heightened oxidative phosphorylation and fatty acid oxidation, is also mediated by upregulated PGC1α expression and facilitated by increased CREB phosphorylation after Crizotinib exposure (14, 114). The mTORC1 pathway, crucial for cell growth and proliferation in GBM (115), is often activated by epidermal growth factor receptor (EGFR). However, mTORC1 inhibition, accompanied by reduced PGC1α expression, protects GBM cells from hypoxia-induced cell death under the conditions of the TME (116, 117). Thus, preclinical experiments have shown that, rapamycin, an mTORC1 inhibitor, triggers adverse effects by promoting cell survival in GBM under hypoxic conditions (116). Concurrently, mTORC1 activation, followed by increased PGC1α expression, sensitizes GBM cells to hypoxia-induced cell death (116).

Similar to other tumors, PGC1α exhibits dual effects in GBM, displaying both anticancer and pro-cancer roles in distinct subtypes. In particular, the fusion of the FGFR3 and TACC3 genes (F3-T3), which act as potent oncogenes, has been identified in approximately 3% of GBM cases (118, 119). PGC1α has been shown to be notably overexpressed in F3-T3-positive GBM cells in the presence of PIN4. Elevated PGC1α contributes to mitochondrial biogenesis and respiration through ERRγ. Conversely, dampening PGC1α activity hinders the tumor-promoting effects of F3-T3, as demonstrated in both cellular and animal models in GBM (120).

### PGC1α in melanoma

Melanoma cells exhibit two distinct transcriptional signatures, proliferative and invasive, which correspond to different cellular phenotypes (121). The metastatic spread of melanoma is thought to involve a transition in cell behavior, shifting from a proliferative program to acquiring migratory and invasive characteristics (122). The expression of PGC1α generally defines these two subsets of melanoma cells (123). In the first subset, PGC1α has been found to be expressed at high levels and plays an important role in melanoma progression and survival. Its upregulation may be triggered by the microphthalmia-associated transcription factor (MITF) via its binding to the upstream regulatory promoter (5, 123, 124), an event regulated by the Wnt/β-Catenin pathway (125) or an important lipogenic enzyme-ATP-citrate lyase (ACLY) (126). Elevated levels of PGC1α are correlated with poor survival (13, 123). In this subset, PGC1α supports melanoma through various mechanisms, with programmed cell death being key. Apoptosis, a process that triggers cell death, is regulated by PGC1α through the regulation of reactive oxygen species (ROS) levels. Thus, suppression of PGC1α leads to a decrease in the expression of genes involved in ROS detoxification, resulting in elevated ROS levels and subsequent induction of apoptosis (123). Ferroptosis, another form of cell death, is involved in melanoma progression and chemoresistance (127). Small molecules that induce ferroptosis, such as RSL3 and ML162, suppress the expression of PGC1α through the Wnt/β-Catenin-MITF pathway. Loss of PGC1α impairs mitochondrial function and antioxidant capacity, leading to excess accumulation of mitochondrial ROS and sensitizing cells to ferroptosis (125). As the activation of the Wnt/β-Catenin pathway in melanoma guides resistance to anti-PD-L1/anti-CTLA-4 treatment (128, 129), targeting the Wnt/β-Catenin signaling pathway or PGC1α may improve the effectiveness of immunotherapy by inducing ferroptosis (129). Furthermore, PGC1α tightly interacts with ERRα in melanomas, promoting mitochondrial oxidative metabolism by regulating the expression of genes involved in oxidative phosphorylation and the TCA cycle (13). Depletion or pharmacological inhibition of ERRα selectively inhibits the growth of PGC1α-positive melanomas, but not PGC1α-negative melanomas (13). BAY 1238097, a potent inhibitor of BET binding to histones, strongly represses the expression of PGC1α in melanoma cells, impairing mitochondrial function and inhibiting melanoma cell proliferation (130). These findings support the concept that PGC1α-positive melanomas depend on mitochondrial metabolism for growth.

Conversely, another subpopulation of melanoma cells exhibits lower PGC1α expression, possesses a limited number of mitochondria, and relies heavily on glycolysis to produce energy. This phenotype is often observed in invasive and metastatic melanomas (131, 132). In this subset of melanoma cells, PGC1α may be epigenetically silenced through chromatin modifications involving H3K27 trimethylation at its promoter. Pharmacological inhibition of EZH2, an enzyme involved in chromatin modifications, diminishes H3K27me3 markers (133, 134), leading to increased PGC1α level and suppression of invasion in PGC1α-silenced cells (122). Additionally, BRAF mutation (V600E) suppresses MITF and PGC1α expression in melanoma cells (135). Knocking down PGC1α in these cells promotes a pro-metastatic gene program and enhances metastasis in mice models (131). PGC1α upregulates the expression of inhibitor of DNA binding protein (ID2), which binds and inhibits a diverse array of bHLH transcription factors (136). The binding of ID2 suppresses the transcription factor TCF4, resulting in the suppression of metastasis-related genes including integrins, which are known to affect metastasis (131, 137). Moreover, ID2 suppresses the activity of TCF12, which increases the expression of WNT5A (122). As WNT5A can stabilize YAP protein levels (138, 139), inhibition of TCF12, WNT5A, or YAP blocks melanoma migration and metastasis (122). BRAF inhibitors, such as PLX4032, which have been reported to upregulate PGC1α expression in melanomas (140, 141), inhibit metastasis partly by suppressing the Wnt/β-Catenin-MITF pathway and promoting the expression of PGC1α (125). This effect is independent of their cytotoxic or growth-inhibitory properties (131). Kisspeptin-1 (KISS1) functions as a metastasis suppressor by inhibiting metastasis without affecting primary tumor growth (142). In melanoma cells, the transcriptional coactivator PGC1α plays a crucial role in mediating the effects of KISS1 on cell metabolism and metastasis suppression (143). PGC1α helps KISS1 upregulate genes that promote fatty acid oxidation, activates AMPK signaling to inhibit acetyl-CoA carboxylase (ACC), and ultimately shifts cells towards mitochondrial oxidative phosphorylation instead of glycolysis (144). The loss of PGC1α blunts these metabolic changes and abolishes KISS1’s anti-metastatic effects. The major implication of these bi-signatures is that effective melanoma therapies should target both proliferative and invasive cell types, as they coexist within tumors and can interconvert. Targeting only one phenotype may lead to the selection and outgrowth of alternative phenotypes. Indeed, suppressing of PGC1α-dependent oxidative metabolism activates glycolysis via HIF1α as a compensatory survival mechanism in melanomas. Dual inhibition of PGC1α and HIF1α causes energetic deficits, but partial rescue of melanoma cells have been observed through glutamine utilization (145). Hence, a triple targeting approach involving PGC1α, HIF1α, and glutamine metabolism is necessary to completely block melanoma growth by shutting down oxidative metabolism, glycolysis, and glutaminolysis (145), suggesting that a combination therapy targeting multiple nodes of tumor metabolism is necessary to effectively disrupt energy production and viability, However, overcoming the challenges posed by metabolic heterogeneity and redundancy remains a significant obstacle.

### PGC1α in prostate cancer

The expression of PGC1α has generally been found to be reduced in PC, with a further decrease observed in metastatic tissues (146). This downregulation of PGC1α is associated with decreased disease-free survival (DFS) (147–149). The exact reasons for the downregulation of PGC1α in PC are not fully understood; however, they are believed to be a result of selective pressure during disease progression and metabolic changes. Reports suggest that miRNAs, such as miR-34a-5p, can downregulate PGC1α (150).

It has been reported that PGC1α plays a tumor-suppressor role in the development of PC, inhibiting cancer progression and metastasis (146). Interestingly, some studies have found that the protein level of PGC1α is undetectable in PC cell lines, despite comparable transcript levels to metastatic PC specimens (146, 151). The re-expression of PGC1α *in vitro* and *in vivo* has been shown to inhibit cell proliferation and cell cycle progression, supporting its antiproliferative activity (146). Moreover, PGC1α suppresses the metastatic properties of PC cells by decreasing integrin signaling, causing cytoskeletal changes (152), and downregulating MYC levels and activity (153). This effect is mediated by its interaction with the transcriptional partner estrogen-related receptor alpha (ERRα). Knockout of ERRα prevents PGC1α from inhibiting invasion, suggesting that the PGC1α/ERRα axis acts as an antagonist to the progression of PC metastasis (146, 152). Furthermore, AMPK, a metabolic regulator in PC, safeguards against cancer progression in mice models (154–156). Activation of AMPK leads to increased expression of PGC1α and its downstream targets, promoting a switch to a more oxidative and catabolic metabolism and opposing the pro-tumorigenic program of increased lipogenesis (154). However, it has been found that androgens-activated AMPK can increase the expression of PGC1α, promoting mitochondrial content and PC cell growth in cell line models (151). Intriguingly, in a mouse model of benign prostatic hyperplasia, androgen/testosterone increased prostate size but did not affect PGC1α levels (151). These findings elucidate the complex roles of the AMPK/PGC1α axis in PC development.

In a subpopulation of clinical PC samples, PGC1α level is found to be overexpressed, and PGC1α may therefore exert a tumor supporting role (151, 157). In addition to the aforementioned AMPK signaling pathway, another mechanism contributing to the abnormal expression of PGC1α is the loss or mutation of p53 (158). In PC cells with mutated or deleted p53, PGC1α has been found to be expressed at high levels. Overexpression of wild-type p53 in these cells decreases the expression of PGC1α and causes mitochondrial dysfunction (157). However, this regulation axis is highly metabolic-pattern dependence, as p53 suppresses PGC1α level and nuclear localization through redox modification (159). In these settings, the tumor-supporting role of PGC1α is found to depend on the transcription factors (TFs) it partnered with. For example, PPARG activation results in the upregulation of AKT3, which subsequently promotes the nuclear localization of PGC1α. The genes induced by PGC1α promotes mitochondrial biogenesis and energy metabolism, fueling PC progression (160).

In addition, the ETS-related gene (ERG) functions as an oncogenic transcription factor in PC (161). In such cases, PGC1α has been shown to act as a coactivator for ERG, specifically under metabolic stress conditions like glucose deprivation and serum starvation (8). This interaction and coactivation of ERG by PGC1α leads to increased expression of antioxidant genes, such as SOD1 and TXN, which can help clear ROS and benefit PC growth (8).This suggests that PGC1α allows ERG fusion-positive PC cells to adapt and survive under metabolic stress by coactivating the antioxidant transcriptional program of ERG.

### PGC1α in ovarian cancer

While PGC1α activity is typically low in normal tissues, several studies have reported frequent overexpression of PGC1α in ovarian tumors compared to that in normal ovaries (162, 163). However, it is important to note that the results of the high tumor expression of PGC1α only correlates with tumor differentiation and did not exhibit significant correlations with other clinical features (164). When combined with ERRα, the overexpression of PGC1α reveals a tendency towards increased risk of metastasis and reduced OS (163). Additionally, the expression of both PGC1α and PGC1β has allowed for the classification of ovarian cancer (OC) patients into distinct subgroups. Approximately 25% of studies tumors exhibits high expression of both genes (164), indicating the presence of an overactive mitochondrial gene program. These tumors demonstrates increased mitochondrial content, oxidative metabolism, and OXPHOS (164). Mechanistic studies have shed light on how the aberrant activation of PGC1α contributes to OC progression and therapeutic resistance. Recent studies have identified PGC1α as a critical driver of OC progression, particularly in high-grade serous OC (HGSOC), which exhibits metabolic heterogeneity (165–167). In OC, the high-OXPHOS state has been linked to chronic oxidative stress (165). This stress leads to the increased aggregation of PML nuclear bodies, which subsequently activates PGC1α through deacetylation. As a result, PGC1α induces the expression of electron transport chain (ETC) components, enhancing mitochondrial respiration in high-OXPHOS cancer cells. Knockdown of PGC1α reduces both ETC gene expression and oxygen consumption rate in these cells (165). Furthermore, PGC1α plays a pivotal role in mediating the response to conventional chemotherapies. PGC1α has been found to be a key regulator of reactive ROS production (165), which are crucial determinants of the apoptotic response to cisplatin in OC cells (168). Elevated expression or activity of PGC1α is correlated with enhanced chemosensitivity by promoting mitochondrial oxidative metabolism and respiration (165). Conversely, reducing PGC1α activity and levels decreases sensitivity to chemotherapy in OC.

### PGC1α in nasopharyngeal carcinoma

There is increasing evidence that metabolic reprogramming driven by PGC1α promotes NPC progression and resistance to treatment. PGC1α has been found to be upregulated in NPC and its high expression has been associated with shorter OS after radiation therapy (169). PGC1α contributes to NPC cell survival by activating FAO pathways, which provide cells with ATP and the antioxidant NADPH. These metabolic alterations allow NPC cells to adapt and thrive under challenging conditions. PGC1α works in conjunction with the transcription factor CEBPB to enhance the expression of CPT1A, a gene involved in FAO, thereby sustaining this metabolic reprogramming (169). Consequently, these changes confer radioresistance to NPC cells (169). Furthermore, TGFβ1, a signaling molecule, can upregulate PGC1α and activate FAO to facilitate EMT and invasion of NPC cells. Specifically, TGFβ1 stimulates phosphorylation and expression of AMPKα1 (170), which, in turn, phosphorylates and activates PGC1α in NPC. This activation leads to transcriptional upregulation of FAO-related genes (170). Inhibiting PGC1α expression and components of the FAO pathway have been shown to reduce EMT, invasion, and metastasis of NPC both *in vitro* and *in vivo*.

### PGC1α in breast cancer

Overall, PGC1α expression has been found to be reduced in breast tumor tissues compared to that in the normal breast epithelium (171, 172). This downregulation of PGC1α potentially facilitates the Warburg effect, in which cells increase their dependence on glycolysis and glucose uptake, while decreasing mitochondrial oxidative phosphorylation, even when oxygen is available (173). Such metabolic shifts enhance the proliferation and survival of cancer cells. A key mechanism that drives this shift is the regulation of mitochondrial deacetylase SIRT3 (171, 174). Although the exact cause of PGC1α’s downregulation in BC cells is yet to be fully elucidated, certain epigenetic modifications such as negative regulation by miR-485 and miR-217 have been proposed (175, 176). Interestingly, despite its general downregulation in breast tumors, the expression of PGC1α varies according to tumor subtypes and their metastatic tendencies. Specifically, HER2^+^ and triple-negative breast tumors (TNBT) express high levels of PGC1α (177, 178). Moreover, elevated expression of PGC1α has been detected in BC cells that predominantly metastasize to the lungs or bone, as opposed to the liver and brain (179). Similarly, circulating tumor cells (CTCs) released from BC in mice models and patients exhibit elevated PGC1α expression (180). Indeed, PGC1α knockdown in a metastatic cell line has been found to result in reduced CTC numbers and metastasis, whereas overexpression of PGC1α has been found to increase lung metastasis *in vivo (* 179, 180). Interestingly, BC cells with low PGC1α levels possess increased metastatic ability when overexpressing PGC1α levels (180). It is worth noting that inhibiting mitochondrial respiration with biguanides in such cells is not found to mitigate PGC1α-induced metastasis (179), suggesting that the augmented metastatic phenotype is not simply attributed to the PGC1α-induced escalation in oxidative phosphorylation. Instead, PGC1α increases overall bioenergetic capacity and flexibility to facilitate metastasis, allowing cancer cells to cope with energy disruptors (179). In these conditions, the induced PGC1α ensures the metabolic demands of aggressive breast tumors.

Early research has also highlighted PGC1α’s involvement in the initiation of BC (181). In particular, its interaction with EglN2, an enzyme involved in the regulation of the hypoxia-inducible factor (HIF) pathway, appears to be central to the modulation of mitochondrial function and has been implicated in BC tumorigenesis (182, 183). In both normoxic and hypoxia conditions, EglN2 forms a complex with both PGC1α and NRF1, leading to the induction of FDXR. This maintains mitochondrial function and contributes to breast tumorigenesis in an HIF-independent manner (182). Importantly, in the absence of PGC1α, the effects of EglN2 overexpression on BC cells are blocked.

Furthermore, PGC1α’s metabolic regulatory functions in BC often operate in collaboration with other transcription factors like ERRα or p53. For instance, the interplay between PGC1α and ERRα governs a spectrum of metabolic genes (172), driving increased mitochondrial respiration, ATP production, and other processes that culminate in heightened tumor aggression and drug resistance in BC (177, 184, 185). In ERBB2^+^ cancer cells, PGC1α positively regulates glutamine metabolism in conjunction with ERRα (177). This regulation contributes to increased glutamine uptake, increased flux through the citric acid cycle (CAC), and enhanced lipogenesis from glutamine, particularly under hypoxic conditions (177). The AMPK orchestrates this energy-sensor axis of PGC1α/ERRα (186). When AMPK is activated, PGC1α/ERRα represses folate cycle and one-carbon metabolism, which are vital for sustaining cell growth in cancer cells. Consequently, repression increases the sensitivity to anti-folate therapy (186). It is well established that mutant p53 confers pro-tumorigenic functions in BCs. Notably, as a key downstream of p53, its function is differentially controlled by the codon 72 variant, highlighting the importance of PGC1α as a “gain-of-function” partner of mutant p53 (187).

From a therapeutic point of view, early studies have hinted at the potential benefits of targeting PGC1α in BC treatment. For instance, interventions with vascular endothelial growth factor receptor 2 (VEGFR2) blockade or the AMPK signaling activator, 5-aminoimidazole-4-carboxamide riboside (AICAR), have shown promising shifts in mitochondrial biogenesis and cancer cell behaviors by modulating PGC1α. One study shows that VEGFR2 blockade by Ki8751 leads to increased activity of PGC1α and thereby stimulates the expression of TFAM, which is essential for mitochondrial DNA transcription and replication (188). Subsequent metabolic reprogramming contributes to increased ROS production and apoptosis in BC cells treated with Ki8751 (188). Moreover, AICAR increases PGC1α expression in triple-negative BC (TNBC) cells (189), mediating mitochondrial biogenesis and contributing to a reduced pro-tumor phenotype and increased chemosensitivity (189). Compound 11, a novel inverse agonist targeting ERRα (190), disrupts ERRα binding to its coactivator PGC1α, with promising anti-tumor activity against triple-negative BC cells and tumors (190). The use of polyethylene glycol-modified graphene oxide (PEG-GO) also results in the selective suppression of PGC1α in cancer cells (191). The reduced ATP production impairs the assembly of the F-actin cytoskeleton and formation of lamellipodia, consequently inhibiting the migration and invasion of metastatic BC cells (191). Importantly, the induction of PGC1α guides drug resistance in the course of chemotherapy of BC (5). Endocrine-resistant BC cells have shown higher PGC1α expression than the parental sensitive lines. PGC1α sensitizes BC cells to low estrogen levels during estrogen deprivation therapy (192–194). This may be an early adaptive response to endocrine therapy that potentially contributes to the development of chemoresistance over time by allowing estrogen hypersensitivity (192). Therefore, inhibiting PGC1α with SR-18292 prevents the growth of therapy resistant cell lines in a dose-dependent manner, while re-expression of PGC1α increases the viability of resistant cells when treating with certain endocrine therapies, such as tamoxifen, fulvestrant, palbociclib, or aromatase inhibitors (193).

## Implications of PGC1α in the tumor microenvironment

The tumor microenvironment (TME) is a complex and dynamic landscape where cancer cells interact with, including immune cells, fibroblasts, and the extracellular matrix. The role of PGC1α in the TME is pivotal yet underexplored. Its involvement goes beyond mere energy metabolism, extending to modulating immune responses and influencing tumor progression and therapy resistance.

Significant insights have been gathered from studies on T cells. Naive T cells normally have high levels of PGC1α, which support their metabolic demands for proliferation and effector functions through mitochondrial biogenesis and oxidative metabolism. However, during T-cell activation, PGC1α expression is progressively repressed (195, 196). Notably, one study observes that, although the mRNA expression of PGC1α in memory CD8^+^ T cells decreases upon activation, its protein expression increases (197). This suggests that specific post-translational mechanisms may regulate the stability of PGC1α in CD8^+^ T cells. In melanomas, tumor-infiltrating T cells have shown a loss of PGC1α level due to the chronic AKT signal activation (195). Additionally, exhausted T cells, experiencing continuous stimulation and hypoxia increase expression of Blimp-1, which further suppress PGC1α expression (196). This impairs their adaptive metabolic responses to hypoxia via mitochondrial biogenesis. Of note, overexpressing PGC1α in these cells enhances their persistence and recall responses, particularly improving the central memory T cell formation and sustained metabolic fitness upon re-exposure to infections (197). Interestingly, the co-stimulatory molecule 4-1BB, which is abundantly expressed in exhausted T cells, promotes mitochondrial biogenesis, fusion, and respiratory capacity (198–200). Costimulation with 4-1BB elevates PGC1α levels, mediating the metabolic effects of 4-1BB signaling (199). Without PGC1α, 4-1BB agonists are less effective at enhancing mitochondrial function and improving anti-tumor responses, or enhance adoptive T cell therapy (199). Thus, restoring the PGC1α expression in functional T cells could offer a strategy to reprogram metabolism in tumor-infiltrating T cells and boost their anti-tumor activity.

Research also shows that PPARγ is essential for maturation of alternatively activated macrophages, enabling monocytes to differentiate into M2 macrophages (201, 202). Indeed, the expression of PGC1α is elevated in these macrophages (203). In breast cancer, a reduced level of miR-382 maintains PGC1α expression in tumor-associated macrophages (203), facilitating the induction of the M2 type through the PPARγ signaling pathway. Fibroblasts also respond to regulation by PGC1α. A recent study found that knocking down PGC1α in normal human lung fibroblasts reduces mitochondrial mass and function (204). This alteration increases activation of matrix synthetic fibroblasts along with secretion of soluble profibrotic factors (204). In mouse models, the loss of PGC1α in induced mouse embryonic fibroblasts (iMEFs) leads to a more aggressive and metastatic melanoma phenotype (205). Similarly, lower PGC1α expression in cancer-associated fibroblasts (CAFs) of oral squamous cell carcinoma (OSCC) enhances the proangiogenic phenotype of CAFs through the PGC1α/PFKFB3 axis (206). Moreover, PGC1α impacts mesenchymal stromal cells (MSCs) (207). In melanoma, cancer cells attract MSCs to the tumor site and induce mitochondrial biogenesis by upregulating PGC1α (207). Furthermore, PGC1α controls mitochondrial transfer from MSCs to melanoma cells, thereby supporting melanoma growth (207).

## Discussion

**Figure 2:**
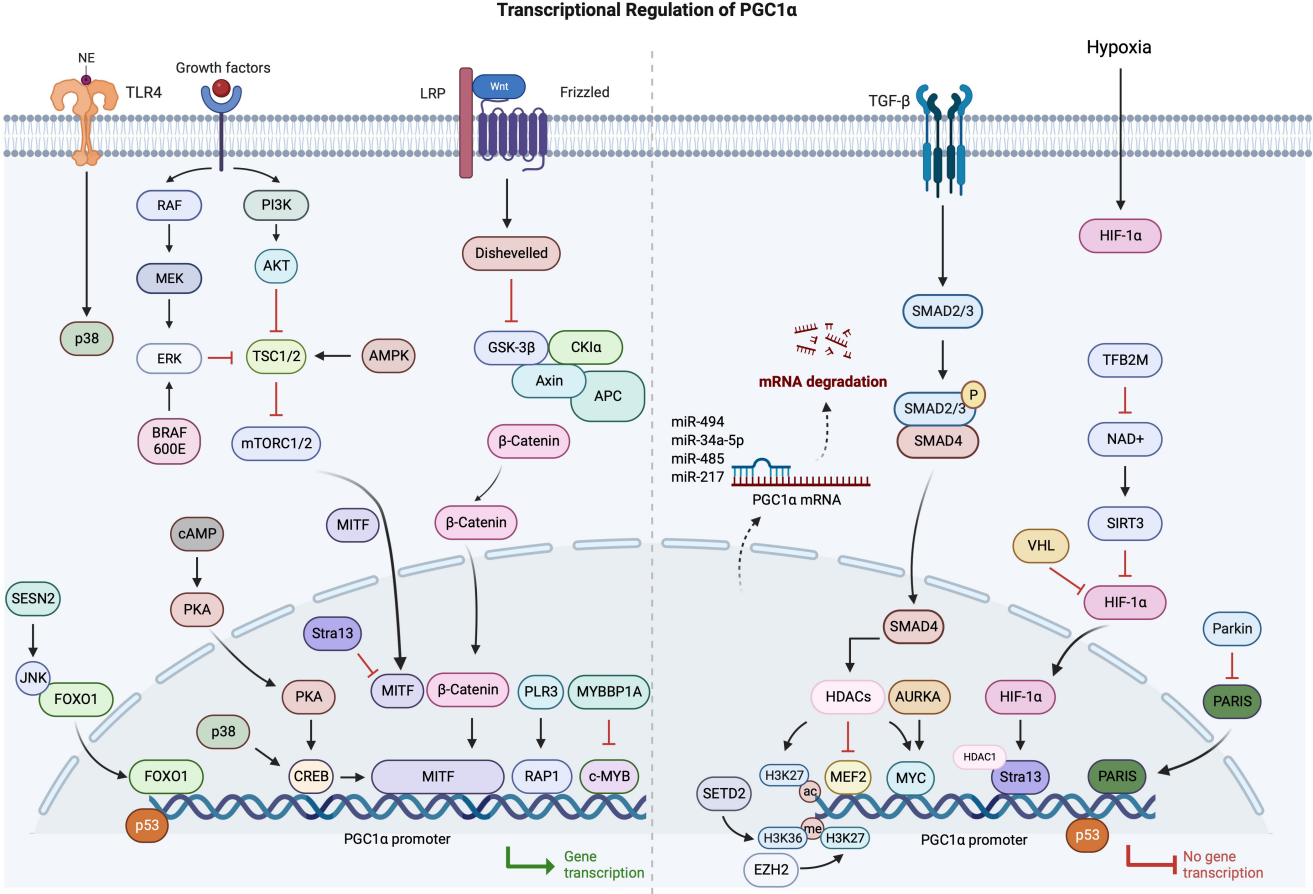
Schematic overview of transcriptional regulation of PGC1a in cancers.

PGC1α is rapidly establishing itself as an indispensable regulator of cancer cell metabolism across numerous malignancies. In cancers, multiple mechanisms are involved in the abnormal expression of PGC1α, particularly in the transcriptional regulation. Therefore, based on the current research progress, we have summarized the relevant findings (**Figure 2**).

Although the expression and functions of PGC1α are context-dependent, it primarily serves as a pivotal orchestrator of mitochondrial biogenesis, oxidative metabolism, antioxidant defenses, and other cellular processes. When PGC1α expression is downregulated, the Warburg effect is facilitated, leading to disease advancement. Subsequent metabolic aberrations can be rectified by reinstating PGC1α levels. This may halt cell proliferation and invasion, and make cells more receptive to chemotherapy. In contrast, PGC1α upregulation promotes oxidative phosphorylation, allows cancer cells to adapt to stress, and promotes a more aggressive cancer phenotype. This duality in biological behavior shows PGC1α’s adaptability in aligning with various co-regulators and executing functions tailored to its environment. Thus, to target PGC1α effectively, understanding its nuanced role in each cancer subtype is indispensable.

Central to PGC1α’s operations is its position at the crossroads of several pivotal signaling pathways involved in cancer. It processes signals from the Wnt/β-catenin, TGF-β, AMPK, AKT, and p53 pathways to regulate downstream metabolic activities. By partnering with transcription factors like ERRα, NRF1, and YAP, PGC1α can drive specific changes in gene expression. Moreover, post-translational modifications such as phosphorylation and acetylation offer another layer of control over its activity. Decoding these intricate molecular ties is a critical step in comprehending PGC1α’s multifaceted functions and how they may go awry in cancer. An intriguing development is the increasing evidence of PGC1α’s profound effect on the tumor microenvironment, particularly its interaction with immune cells.

Strategic targeting of PGC1α in cancer therapy, therefore, requires a nuanced approach that considers its dual functionality. In cases where PGC1α functions as a tumor suppressor, its upregulation or enhanced activity can shift cancer cell metabolism away from the Warburg effect. This metabolic shift involves reducing glycolysis and increasing oxidative phosphorylation, which typically slows cancer progression and may make cancer cells more amenable to interventions that induce metabolic stress. Enhancing PGC1α’s expression could be achieved through gene therapy techniques, and small molecule activators. Conversely, in cancers where PGC1α contributes to a more aggressive phenotype, its function is linked to enhanced oxidative phosphorylation, supporting cancer cell adaptation to metabolic and oxidative stress. In such cases, inhibiting PGC1α might reduce the cancer cells’ ability to sustain high energy demands and resist hostile environments, such as those imposed by chemotherapy. This can be approached through the use of small molecule inhibitors that disrupt PGC1α’s interaction with its coactivators or transcription factors it regulates. Additionally, RNA interference technologies could selectively knock down PGC1α mRNA, diminishing its protein levels and thus its functionality in cancer cells. Both strategies—enhancing or inhibiting PGC1α—must consider the cancer type, the specific metabolic profile of the tumor, and the systemic implications of altering metabolic pathways. For instance, enhancing oxidative metabolism in non-tumor cells might also affect normal cells, leading to unintended consequences like increased reactive oxygen species. Similarly, inhibiting PGC1α in aggressive tumors must be carefully managed to avoid crippling normal cells’ ability to manage oxidative stress. Effective therapeutic strategies should aim to disrupt this metabolic adaptability by targeting PGC1α along with its regulatory network to block compensatory pathways that facilitate resistance to therapy. Furthermore, PGC1α’s impact on the tumor microenvironment, particularly through its influence on the metabolic states of T cells and macrophages, is gaining attention. By modulating immune cell metabolism, PGC1α could potentially alter the immunological landscape of tumors, reducing immune suppression and enhancing the efficacy of immunotherapies. This understanding suggests that strategies which leverage PGC1α’s role in the tumor microenvironment could complement direct targeting approaches, creating a multifaceted attack on tumor growth.

In summary, PGC1α’s multifaceted roles in cancer metabolism indicate that it is a promising therapeutic target for cancer. Developing drugs that can specifically modulate PGC1α’s activity, tailored to the unique metabolic profiles of different cancer types, represents a promising approach in oncology. By doing these, we are now on the brink of translating our understanding of this metabolic mediator into its clinical benefits against cancer.

## Author contributions

YW: Writing – review & editing, Writing – original draft, Investigation. JP: Resources, Methodology, Writing – review & editing, Writing – original draft. DY: Software, Writing – review & editing, Writing – original draft. ZX: Validation, Writing – review & editing, Writing – original draft. BJ: Resources, Writing – review & editing, Writing – original draft. XD: Methodology, Writing – review & editing, Writing – original draft. CJ: Investigation, Conceptualization, Writing – review & editing, Writing – original draft. BO: Visualization, Validation, Methodology, Data curation, Writing – review & editing, Writing – original draft, Funding acquisition. LS: Supervision, Software, Resources, Project administration, Investigation, Funding acquisition, Formal analysis, Conceptualization, Writing – review & editing, Writing – original draft, Visualization, Validation, Methodology, Data curation.

## Glossary

**Table:**

PGC1α	Peroxisome proliferator-activated receptor gamma coactivator 1-alpha
PPARγ	Peroxisome proliferator-activated receptor gamma
TCGA	The Cancer Genome Atlas Program
AMPK	AMP-activated protein kinase
CRC	Colorectal cancer
SIRT3	NAD-dependent deacetylase sirtuin-3
shRNA	Short hairpin RNA
PRL3	Phosphatase of regenerating liver 3
CSCs	Cancer stem cells
TME	Tumor microenvironment
NET	Neutrophil extracellular trap
NE	Neutrophil elastase
TLR4	Toll-like receptor 4
EVs	Extracellular vesicles
ROS	Reactive oxygen species
FASN	Fatty acid synthase
Sp1	Specificity protein 1
SREBP-1c	Sterol regulatory element-binding protein 1
AKT	Protein kinase B
GSK-3β	Glycogen synthase kinase-3 beta
LARS1	Leucyl-tRNA synthetase 1
EMT	Epithelial–mesenchymal transition
BCL2	B-cell lymphoma 2
PARP-1	Poly [ADP-ribose] polymerase 1
SIRT1	NAD-dependent deacetylase sirtuin-1
LA	Linoleic Acid
MH	Manuka honey
GC	Gastric cancer
SNAI1	Snail family transcriptional repressor 1
CAB39L	Calcium binding protein 39 like
HCP5	HLA Complex P5
CEBPB	CCAAT/enhancer-binding protein beta
CPT1	Carnitine palmitoyltransferase 1
FAO	Fatty acid oxidation
HCC	Hepatocellular carcinoma
NASH	Non-alcoholic steatohepatitis
PARIS	Parkin-interacting substrate
SESN2	Sestrin2
FOXO1	Forkhead box protein O1
YAP	Yes-associated protein 1
TFB2M	Mitochondrial transcription factor B2
PDK1	Pyruvate Dehydrogenase Kinase 1
HNF4α	Hepatic nuclear factor 4 alpha
G6PC	Glucose 6-phosphatase alpha
PCK1	Phosphoenolpyruvate carboxykinase 1
GCN5	General control non-depressible 5
HMGA1	High mobility group AT-hook 1
NAFLD	Non-alcoholic fatty liver disease
Nrf2	Nuclear factor erythroid 2-related factor 2
ARE	Antioxidant response element
TIGAR	TP53 induced glycolysis regulatory phosphatase
PSAT1	Phosphoserine aminotransferase 1
TFAM	Mitochondrial transcription factor A
OXPHOS	Oxidative phosphorylation
TCA	Tricarboxylic acid cycle
RCC	Renal cell carcinoma
ccRCC	Clear cell renal cell carcinoma
TGF-β	Transforming growth factor beta
HDAC7	Histone deacetylase 7
Stra13	Retinoic acid 13
FTO	Fat mass and obesity associated
DDR1	Discoidin domain receptor family, member 1
MPC1	Mitochondrial pyruvate carrier 1
ERR-α	Estrogen-related receptor alpha
MYBBP1A	MYB binding protein 1A
pVHL	Von Hippel–Lindau tumor suppressor
SETD2	SET domain containing 2
CCA	Cholangiocarcinoma
PDHA1	Pyruvate dehydrogenase-alpha 1
GBM	Glioblastoma
PDX	Patient-derived xenograft
TMAs	Tissue microarrays
PTEN	Phosphatase and tensin homolog
AURKA	Aurora kinase A
CHIP	Chromatin immunoprecipitation
ATAC	Assay for transposase-accessible chromatin
CREB	cAMP response element-binding protein
EGFR	Epidermal growth factor receptor
mTORC1	Mammalian target of rapamycin complex 1
FGFR3	Fibroblast growth factor receptor 3
TACC3	Transforming acidic coiled-coil containing protein 3
MITF	Microphthalmia-associated transcription factor
ACLY	ATP-citrate lyase
RSL3	RAS-selective lethal 3
CTLA4	Cytotoxic T-lymphocyte associated protein 4
BET	Bromodomain and extra-terminal domain
EZH2	Enhancer of zeste homolog 2
ID2	Inhibitor of DNA binding 2
TCF4	Transcription factor 4
TCF12	Transcription factor 12
KISS1	Kisspeptin-1
ACC	Acetyl-CoA carboxylase
AMPK	AMP-activated protein kinase
MSCs	Mesenchymal stromal cells
iMEFs	induced mouse embryonic fibroblasts
PC	Prostate cancer
DFS	Disease-free survival
ERG	ETS-related gene
SOD1	Superoxide dismutase 1
CAFs	Cancer-associated fibroblasts
HGSOC	High-grade serous ovarian cancer
ETC	Electron transport chain
NPC	Nasopharyngeal carcinoma
BC	Breast cancer
TNBT	Triple-negative breast tumors
CTCs	Circulating tumor cells
EglN2	Egl nine homolog 2
HIF	Hypoxia-inducible factor
FDXR	Ferredoxin reductase
TAMs	Tumor-associated macrophages
AICAR	5-Aminoimidazole-4-carboxamide riboside
VEGFR2	Vascular endothelial growth factor receptor 2
PEG-GO	Polyethylene glycol-modified graphene oxide. CD147, Cluster of differentiation 147
TACC3	Transforming acidic coiled-coil containing protein 3
HBV	Hepatitis B virus
Blimp-1	B lymphocyte-induced maturation protein-1
ERRγ	Estrogen related receptor gamma.
